# Economic threshold analysis of delivering a task-sharing treatment for common mental disorders at scale: the Friendship Bench, Zimbabwe

**DOI:** 10.1136/ebmental-2021-300317

**Published:** 2021-11-18

**Authors:** Andrew Healey, Ruth Verhey, Iris Mosweu, Janet Boadu, Dixon Chibanda, Charmaine Chitiyo, Brad Wagenaar, Hugo Senra, Ephraim Chiriseri, Sandra Mboweni, Ricardo Araya

**Affiliations:** 1 Health Services and Population Research, King's College London, London, UK; 2 Friendship Bench, Harare, Zimbabwe; 3 Research Support Trust, Department of Community Medicine, University of Zimbabwe, Harare, Zimbabwe; 4 Department of Health Policy, London School of Economics, London, UK; 5 Department of Epidemiology, University of Washington, Seattle, Washington, USA; 6 Center for Research in Neuropsychology and Cognitive and Behavioral Intervention (CINEICC), University of Coimbra, Coimbra, Portugal; 7 School of Health and Social Care, University of Essex, Colchester, UK

**Keywords:** adult psychiatry, anxiety disorders, depression & mood disorders

## Abstract

**Background:**

Task-sharing treatment approaches offer a pragmatic approach to treating common mental disorders in low-income and middle-income countries (LMICs). The Friendship Bench (FB), developed in Zimbabwe with increasing adoption in other LMICs, is one example of this type of treatment model using lay health workers (LHWs) to deliver treatment.

**Objective:**

To consider the level of treatment coverage required for a recent scale-up of the FB in Zimbabwe to be considered cost-effective.

**Methods:**

A modelling-based deterministic threshold analysis conducted within a ‘cost-utility’ framework using a recommended cost-effectiveness threshold.

**Findings:**

The FB would need to treat an additional 3413 service users (10 per active LHW per year) for its scale-up to be considered cost-effective. This assumes a level of treatment effect observed under clinical trial conditions. The associated incremental cost-effectiveness ratio was $191 per year lived with disability avoided, assuming treatment coverage levels reported during 2020. The required treatment coverage for a cost-effective outcome is within the level of treatment coverage observed during 2020 and remained so even when assuming significantly compromised levels of treatment effect.

**Conclusions:**

The economic case for a scaled-up delivery of the FB appears convincing in principle and its adoption at scale in LMIC settings should be given serious consideration.

**Clinical implications:**

Further evidence on the types of scale-up strategies that are likely to offer an effective and cost-effective means of sustaining required levels of treatment coverage will help focus efforts on approaches to scale-up that optimise resources invested in task-sharing programmes.

## Background

Task-sharing approaches offer an evidence-based and pragmatic approach to treating common mental disorders (CMD) in low-income and middle-income countries (LMICs).^1 2^ The Friendship Bench (FB), developed in Zimbabwe, is one example.^3^ It is a cultural adaptation of problem-solving therapy (PST) delivered by lay health workers (LHWs) in primary care settings. LHWs, typically older female multitasking practitioners, are trained to deliver up to six sessions of PST. Sessions take place on a discreetly positioned bench within the grounds of the health clinic. Patients then have the option of attending a series of peer-led group support meetings. The FB has been tested for its efficacy in a randomised controlled trial and shown to improve clinical outcomes at 6-month follow-up.^4^ There has since been a major scale-up of the FB beginning in 2016 across Harare, Gweru and Chitungwiza,^5^ covering 36 primary care sites.

Few studies have evaluated the implementation of treatments for CMD at scale,^6^ and none has considered whether scale-up of a task-sharing treatment model into routine care settings would be a cost-effective use of resource. Several studies have examined the cost-effectiveness of specific task-sharing approaches, combining evidence from clinical trials on service user outcomes, incremental treatment costs and impact on wider service utilisation.^1 2 7–9^ However, they do not consider the broader question of whether scale-up beyond research settings into routine care is a cost-effective investment. This requires quantification of all fixed costs involved with delivering a programme at scale, including the cost of resources allocated to the implementation of the scale-up and the infrastructure required to sustain delivery year-on-year. It is also dependent on the level of treatment coverage achieved, treatment effectiveness outside of trial settings and the cost of treatment delivered in routine practice.^10 11^

## Objective

We undertook a threshold analysis to assess the level of treatment coverage needed for a scale-up of the FB recently implemented in Zimbabwe to be considered cost-effective investment.

## Methods

### Analytical approach

The threshold analysis was undertaken within a ‘cost-utility’ framework with treatment benefit quantified as the avoidance of years lost due to disability (YLD)^12^ associated with CMD. The YLD measure forms part of the disability-adjusted life year (DALY) approach to estimating disease burden and treatment impact.^12^ We chose this metric to capture treatment benefit because it has a wide usage in economic evaluations carried out in a global health context.^12^ DALY is conventionally defined as the sum of years of life lost due to premature death and the YLD attributable to CMD. We focus on the YLD component as a measure of treatment benefit given uncertainty over the direct causal component of a substantial proportion of the excess mortality linked to CMD.^13^

Modelling was undertaken to estimate the YLD avoided through treating CMD using the FB rather than a usual care comparator. This used evidence and data on treatment effect and treatment contacts from the FB clinical trial described elsewhere.^4^ We use this single source of evidence given that the trial was conducted within the same geographical and service-related context within which the wider scale-up of the FB took place. Usual care was assumed to comprise the type and frequency of health professional contacts self-reported by participants allocated to the control group of the trial. We estimated YLD over a 2-year time horizon to avoid uncertainty with projections of service user outcome over lengthier periods. Following convention, YLD in year 2 are discounted at a recommended rate of 3%.^12^ Costs are quantified from a payer perspective: 70%–80% of the FB programme, including scale-up, has been funded through non-governmental finance, with the remainder resourced from local city health department budgets.

We identify the level of treatment coverage (annual number treated) required for the investment in the scale-up of the FB to be considered cost-effective based on a prespecified cost-effectiveness threshold (CET). We refer to the cost-effective treatment coverage as the ‘number needed to treat’ (NNT). To evaluate the NNT, the annual fixed costs of delivering the FB programme in Zimbabwe were estimated inclusive of resource inputs invested in the initial implementation of the scale-up and programme infrastructure required to sustain the programme year-on-year (excluding the variable costs of clinical assessment and treatment-related activity with service users). We then convert these fixed costs into their ‘opportunity cost’ equivalent (C)—the quantity of YLD that could have been averted had the resources subsumed within the programme’s fixed costs been invested in alternative health promotional activity. This is calculated as:

$$C=\frac{Fixedprogrammecost}{\lambda }$$

where ‘ λ ’ is a CET appropriate for Zimbabwe. The CET is intended to approximate the additional dollar expenditure on healthcare inputs sufficient to produce a one-unit reduction in disease burden, thereby indicating the maximum a health system should be willing to pay to avert a single YLD.^14^ We adopt a CET of US$600 per YLD averted, equivalent to 50% of the gross national income (GNI) per capita in Zimbabwe at 2019 price levels.^15^ This follows the recommendations on threshold determination in LMIC settings, reflecting the principle of opportunity cost and affordability within resource-poor contexts.^16 17^ The value of ‘C’ is relevant to this analysis because it identifies the minimum quantity of annual treatment benefit (total YLD averted) the FB would need to generate compared with usual care to justify fixed costs. The NNT value required for cost-effective scale-up is then:

$$NNT=\frac{C}{INB}$$

where ‘INB’ is the incremental net benefit per service user of FB treatment, equal to the YLD avoided through replacement of usual care with the FB less the opportunity cost of additional LHW time inputted to FB treatment-related activity: clinical assessments, PST sessions, indirect costs (defined below), case assessment work and peer group attendance. The opportunity cost of treatment activity is again expressed as the YLD that would otherwise be averted (if LHW time was used elsewhere) and is estimated using the same method applied to fixed costs.

In addition to the NNT we also report the incremental cost-effectiveness ratio (ICER) for the FB programme (additional cost per YLD averted):

$$ICER=\frac{\left(\$totalfixedprogrammecostperpatient+\$incrementaltreatmentcostperpatient\right)}{YLDavertedperpatient}$$

The base case ICER is calculated assuming an annual level of treatment coverage equivalent to the recorded number of patients seen by the FB during 2020 (obtained from programme management information).

A Markov model was used to estimate the YLD that could be avoided if a cohort presenting with CMD received FB treatment in place of usual care. A Markov approach was selected because it is amenable to projecting service user outcomes over extended time horizons.^18^ Outcomes are simulated over 24 1-month cycles for FB and usual care treatment scenarios. For simplicity the analysis only considers outcomes relating to a single treatment episode.

A visual description of the model is provided in the online supplemental appendix. In summary, the model assumes that service users spend time in one of two health states characterised by a unique disability weighting: a CMD and a remission state. Disability weights (table 1) were obtained by transforming (see table 1 footnote) Zimbabwean-specific ‘utility’ scores applicable to self-reported health states for participants in the FB clinical trial.^4 19^ Health states were identified through administration of the EQ5D-5L health-related quality of life instrument.^20^ Over a series of monthly post-treatment ‘cycles’, a percentage of the model cohort are expected to either transition into the remission state or remain in the CMD state. Of those who remit, a percentage are assumed to relapse back to the CMD state during each cycle, with a further proportion of those who relapse transitioning back to the remission state.

10.1136/ebmental-2021-300317.supp1 Supplementary data

**Table 1:** Modelling assumptions

Treatment effectiveness	
Prevalence ratio for CMD state at 6 months post-treatment (score ≥9 on SSQ-14): FB vs usual care	0.21
% of service users entering remission each month post-treatment*	
FB	28
Usual care	7
**Relapse**	
% of remitters who relapse within 12 months (FB and usual care)	53
Implied % of remitters who relapse each month*	6
% of those who relapse who go back into remission within 12 months (FB and usual care)	49
Implied % of those who relapse who go back into remission each month*	5
**Disability weights†**	
CMD state (score ≥9 on SSQ-14)	0.41
Remission (score <9 on SSQ-14)	0.15
**Mortality, % (FB and usual care)**	0.5
Population monthly survival probability (both sexes)	0.29
Relative mortality risk	1.71
% of treatment cohort who die in each monthly cycle	0.5
**Costs**	
*FB fixed costs*	
*Scale-up costs (US$*)	
Phase 1: needs assessment	$64 751
Phase 2: LHW training	$120 709
Phase 3: implementation	$289 382
Total scale-up cost	$474 842
*Central programme overhead costs (annual; US$*)	
Staff	$251 640
Running costs	$24 024
Building occupied (annuitised cost of capital)‡	$6617
% of central operational costs attributable to FB	40
Total annual operational cost attributable to FB (total operational cost × 40%)	$112 913
*Other programme infrastructure—time input*	
Patient mobilisation by LHWs (hours per clinic per month)	9.20
Oversite from district health promotion officers (hours per clinic per month)	2.00
*FB variable costs*	
*Treatment-related activity*	
Number of clinical assessments undertaken per treatment episode	7.12
LHW face-to-face treatment time with patient (hours per treated service user)	3.35
Additional non-patient contact time spent by LHW on administrative duties associated with each treatment episode (hours per treated patient)	3.35
Time allocated by LHW and LHW supervisors to case review (hours per treated service user)	0.23
LHW attendance at peer group meetings (hours per treated service user)	0.44
**Usual care resource usage**	
Health professional time (number of contacts per treated service user)	
Public hospital doctor§	0.03
Public health clinic doctor§	0.03
Psychiatrist§	0.03
Community health worker¶	0.03
Clinic nurse¶	0.07
Counsellor§	0.18
**Health practitioner unit costs (based on annual salaries)**	
LHW	$0.23 per hour
LHW supervisor	$0.23 per hour
Clinic nurse	$0.99 per hour
District health promotion officer	$0.99 per hour
Community health worker	$0.11 per contact
Psychiatrist	$3.75 per contact
Counsellor	$0.23 per contact
Public doctor (hospital and community)	$1.88 per contact
Clinical specialist	$3.75 per contact
**Discount rate, %**	3

Created by the authors.

*To estimate a monthly % of the cohort who transition from remission to relapse or from relapse back into remission, we take the observed % (P) who have transitioned within the period elapsed (t; 12 months) using the reported values from the relevant papers cited in the main text and convert this to a rate of transition ‘r’ using the formula r=[*−log*(1−P)]/t. The rate is then converted to probability ‘Pr’ (%) using the formula Pr=1−exp(−rt). This method makes the simplifying assumption that the rate of transition from one state to another is constant through time.

†Disability weights (D) are transformed values of Zimbabwean population utility weights (U) applicable to self-reported health states for FB trial participants. Utility scores are located on a scale anchored at 1 (full health) and 0 (death), with negative scores allowed to account for health states viewed as being less preferable to death. The transformation is: D=1−U. This effectively characterises ‘disability’ as a health loss. For example, a disability weight=1 (1 minus U=0) describes a health loss/level of disability equivalent to death; a disability weight=0.1 (1 minus U=0.9) describes a relatively minor health loss/disability level. For modelling, we use the mean derived disability weight for participants at the trial baseline to weight the CMD state (eligible participants were required to have a CMD prior to randomisation); and the mean disability weight for participants identified to be in remission at follow-up (score <9 using the SSQ-14) to weight the remission state.

‡Based on purchase price of property housing central team annuitised assuming a discount rate of 3% and an asset lifetime of 80 years.

§Each contact assumed to use 60 min of health professional time in total, inclusive of patient contact and non-contact time.

¶Each contact assumed to use 30 min of health professional time in total, inclusive of patient contact and non-contact time.

CMD, common mental disorder; FB, Friendship Bench; LHW, lay health worker; SSQ-14, Shona Symptom Questionnaire.

The per cent of service users entering remission during each monthly cycle (table 1) was inferred using the reported proportion of participants with CMD at 6-month follow-up in the FB clinical trial control group combined with the reported prevalence ratio for CMD between intervention and control participants.^4^ The presence of CMD was defined according to whether a trial participant scored ≥9 on the Shona Symptom Questionnaire (SSQ-14), a locally validated assessment tool for CMD used routinely to determine treatment eligibility.^21^ We present an assessment of the impact on the NNT value of using less favourable assumptions regarding CMD prevalence ratios in sensitivity analysis.

The monthly per cent of remitters who relapse (table 1) was estimated using 12-month relapse outcomes reported in a rare example of published research into the duration of remission following low-intensity psychological therapy (in this case cognitive–behavioural therapy delivered in a British primary care service).^22^ Relapse rates for FB treatment and usual care are assumed to be equivalent, an assumption that has been employed in similar economic analysis of depression outcomes in an LMIC setting.^8^ The monthly per cent of further remission after relapse was estimated using evidence from a Zimbabwean observational study that examined remission outcomes for a cohort of cases with a CMD attending community health facilities and traditional practitioners.^23^

Over each modelling cycle a percentage of the cohort are also assumed to die (effectively exiting the model; table 1). This was estimated using annual survival probabilities contained in life tables for Zimbabwe,^24^ adjusted by a relative mortality risk reported for populations with depression.^25^ As our analysis excludes avoidance of years of life lost as a treatment benefit, mortality risk is fixed at the same level for both remission and time spent in a CMD state.

### Costs

All cost-related assumptions are detailed in table 1. Annual fixed costs were obtained from programme-level financial data. The cost of the programme scale-up came from financial planning data for 2016 detailing anticipated expenditures across multiple activities. Data on actual expenditures were unavailable. The FB scale-up strategy consisted of three phases: a needs assessment, LHW training in PST and a final ‘implementation’ phase. Cost estimates relate to the hiring of venues and accommodation, purchase of equipment, transportation, payments for trainer time, training of research assistants and purchase of wooden benches (for PST sessions). Costs were converted to an annual fixed cost equivalent assuming a 10-year programme lifetime and a discount rate of 3%.

Central programme overhead costs included payment for staff involved with programme management and related activities (eg, analytical and administrative support), building space used to house central programme activities and associated running costs. The annual cost of used building space was estimated using the purchase value of the property converted to an annualised cost, applying a discount rate of 3% and an asset lifetime of 80 years. As central overhead costs are shared across other non-FB activities, the central programme team estimated that 40% of overheads would be attributable directly to the FB.

The number of clinical assessments undertaken to determine treatment eligibility for every service user treated was inferred based on fieldwork data received from all clinics, collected as part of wider ongoing research on programme implementation, identifying the mean percentage of patients clinically assessed who had at least one FB session (36%); and an assumed 39% case detection rate through clinical screening as observed within the FB clinical trial.^4^ Each clinical assessment was assumed to require 60 min of LHW time.

The duration of LHW time allocated to PST sessions was estimated using the mean frequency of sessions reported in the FB trial data, assuming 45 min per session. For every minute of LHW direct treatment time, we assumed an additional minute would be required for preparatory and other clinical and administrative tasks (we refer to these as ‘indirect costs’). Time spent by LHW and supervisors reviewing patients was assumed to take an average of 13.5 min per patient. These assumptions were informed by treatment resource requirements reported by Araya *et al*,^1^ in relation to a task-sharing intervention delivered in Chile. Time allocated by LHWs to attendance at peer group meetings was based on data from the FB clinical trial. It was assumed that LHWs would be expected to attend one in every six peer group meetings, with attendance lasting 60 min.

LHWs are expected to engage in patient ‘mobilisation’. This typically consists of a talk given in a clinic waiting area promoting mental health awareness and the FB. Time allocated to mobilisation was estimated based on the mean number of mobilisation sessions over 1 month reported by a sample of LHWs interviewed during fieldwork for wider ongoing research. A group mobilisation talk was assumed to last 15 min. City health department district health promotion officers provide supervisory input to the FB programme. In consultation with programme leads, this was assumed to consist of a weekly 30 min visit to each clinic providing the FB.

The cost of usual care was estimated using health professional contact data self-reported over follow-up by participants in the control group of the FB clinical trial (unpublished data; D.Chibanda et al. (2016)). Assumptions regarding the quantity of time allocated to each contact are found in the footnote to table 1. The cost of LHW and other staff time allocated to the FB and usual care was valued using staff salaries provided by the FB programme.

### Currency value and adjustments for inflation

All costs are reported in US dollars at 2019 price levels. We have followed published guidance^26 27^ on exchange rate adjustment and accounting for inflation (scale-up costs were originally reported in Canadian dollars at 2016 prices).

### Sensitivity analysis

Deterministic sensitivity analyses were undertaken to examine the effect of compromised levels of treatment effect assumed for treatment in routine settings (increasing prevalence ratio for CMD) on the NNT. We also report the NNT using alternative CETs: $300 per YLD averted (50% of the recommended base case value) and $1200 per YLD averted—equating to GNI per capita, the level currently recommended by the WHO for identifying ‘very cost-effective’ health programmes.^28^ We also explore the effect of deviations from base case assumptions on the NNT value for a range of other model parameters. Alternative ICERs are presented in a two-way sensitivity analysis across varying treatment coverage levels and treatment effect.

All analyses were conducted using Microsoft Excel and Stata V.15. We follow published guidance on the reporting of economic evaluations.^29^

## Findings

Estimates of fixed programme costs, costs of treatment, costs of usual care and the incremental benefit of treatment per service user are presented in table 2. The greater proportion of annual programme costs were attributed to a combination of costs of scaling up the FB and the fixed costs of programme infrastructure; treatment-related costs accounted for only a small proportion of the overall resource impact (table 2).

**Table 2:** Costs and treatment benefit

Fixed cost of scale-up (annual equivalent)	$55 666		
Fixed programme infrastructure cost (central overhead cost + cost of service user mobilisation + cost of DHPO input to the programme; annual)	$114 753		
Total fixed programme cost per year	$170 419		
Variable cost of treatment per service user (clinical assessment + treatment sessions + indirect and case review costs + LHW peer group meeting attendance)	$3.37		
Cost of usual care per service user	$0.33		
**Contribution of each cost component to total annual cost of FB**			
Fixed cost of scale-up, %	31		
Fixed programme infrastructure cost, %	63		
Treatment cost*, %	6		
**Programme benefit**			
YLD over 24 months per service user: FB	0.414		
YLD over 24 months per service user: usual care	0.502		
YLD averted per service user due to treatment with FB	0.088		
Incremental net benefit of FB treatment per service user	0.084 YLD averted		
NNT (base case estimate: CET=$600 per YLD averted)	3413 service users		
**One-way sensitivity analysis (varying CET)**	**CET=$300 per YLD averted**		**CET=$1200 per YLD averted**
NNT	7269 service users		1656 service users
**Two-way sensitivity analysis of ICER**	**2020 treatment coverage (base case: 12 364 treated**)	**50% of 2020 treatment coverage (6182 treated**)	**25% of 2020 treatment coverage (3091 treated**)
**Treatment effect=100% of prevalence ratio for CMD observed in FB trial**	$191 per YLD averted	$347 per YLD averted	$659 per YLD averted
50%	$302 per YLD averted	$549 per YLD averted	$1044 per YLD averted
25%	$528 per YLD averted	$961 per YLD averted	$1827 per YLD averted

Created by the authors.

*Annual cost of treatment calculated at the cost-effective NNT value.

CET, cost-effectiveness threshold; CMD, common mental disorder; DHPO, district health promotion officer; FB, Friendship Bench; ICER, incremental cost-effectiveness ratio; LHW, lay health worker; NNT, number needed to treat; YLD, year lived with disability.

The NNT required for programme cost-effectiveness was 3413 service users per year. The ICER for the FB at 2020 treatment coverage levels (12 364 service users seen by the FB over 12 months) was $191 per additional YLD averted. At this level of treatment coverage, the ICER is below the base case CET of $600 per YLD averted.

**Figure 1:**
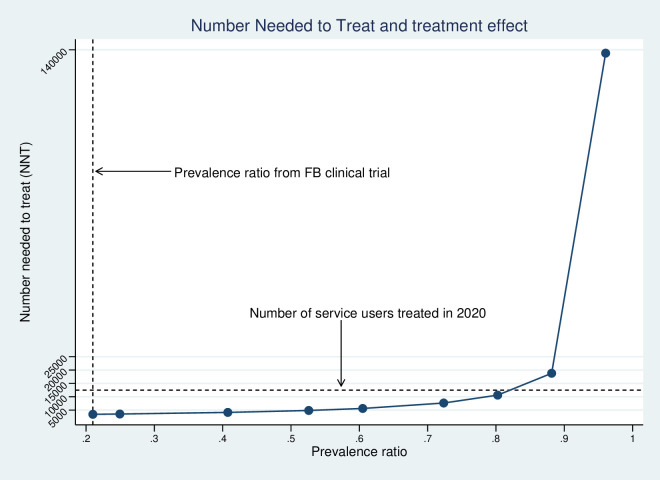
The number needed to treat at varying levels of treatment effect (FB=Friendship Bench)

**Figure 2:**
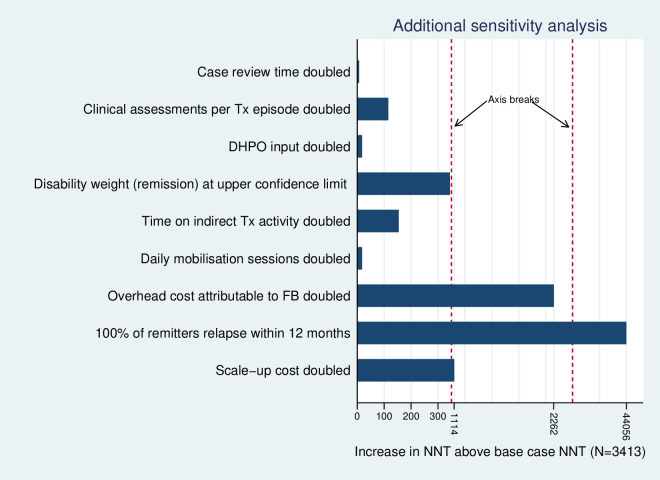
Additional one-way sensivity analysis of model parameter values (Tx=Treatment; DHPO=District Health Promotion Officer;FB=Friendship Bench; NNT=Number needed to treat)

In one-way sensitivity analysis the NNT was relatively insensitive to an increasing prevalence ratio for CMD between FB and usual care treatment: at a ratio above 0.88, the NNT begins to increase substantially (figure 1). Regarding other model parameters, the NNT was most sensitive to an increase in the assumed relapse rate to 100% of remitters relapsing within 12 months (figure 2). As anticipated, the NNT also varies with the chosen CET (table 2). Two-way sensitivity analysis (table 2) of the ICER indicates that scale-up would no longer be cost-effective (ICER >$600 per YLD averted), where treatment coverage is at 25% of the 2020 observed level or treatment coverage is at 50% of the 2020 level combined with a prevalence ratio equating to a treatment effect at 25% of the value observed under clinical trial conditions (prevalence ratio=0.80).

## Discussion

A threshold analysis was used to evaluate whether scale-up of the FB, a task-sharing model for treating CMD in Zimbabwe, was cost-effective. We identified incremental fixed and variable programme costs and the YLD avoided through replacement of usual care with the FB. ‘Usual care’ amounted to very little in terms of active treatment input: only 10% of the participants attending health clinics randomised to usual care in a clinical trial of the FB had any contact with counselling for CMD,^4^ highlighting the pre-existing treatment gap in the localities where FB scale-up occurred.

We estimated that 3413 service users would need to have been treated annually for the scale-up to be cost-effective, equivalent to only 10 per year across 340 trained LHWs known to have been active in delivering the FB. Treatment effectiveness in routine practice may not match that observed within a clinical trial: practitioners may deviate from the intended treatment model and patients may be less engaged. However, even with a significant reduction in effect (a prevalence ratio for CMD of 0.80 compared with 0.21 observed under trial conditions), the required NNT remained modest in magnitude: equivalent to 31 service users treated per active LHW per year (or just under 3 per month). When judged against recorded levels of treatment coverage during 2020 (12 463 service users treated, 37 per LHW per year), this seems a plausible goal, although most treatment contacts during this period took place on an outreach basis outside of health clinics due to the COVID-19 pandemic.

A two-way sensitivity analysis of the ICER estimated at the 2020 treatment coverage level highlights the importance of achieving adequate levels of coverage to mitigate the risk of compromised treatment effect. There has been little research conducted into which types of strategy for implementing scale-up offer the most effective and cost-effective methods of sustaining higher coverage levels in CMD populations.^6^ Further investment in service improvement research would help identify the approaches more likely to optimise use of scarce resources invested in the scale-up of task-sharing treatments. Our analysis also suggests that achievement of a cost-effective scale-up may depend on relapse rates. While we adopted a more extreme assumption to test this (100% relapse within 12 months of remission), higher relapse rates combined with compromised treatment effect could pose a significant risk to achieving a cost-effective outcome. Further research into longer-term outcomes would reduce uncertainty around the level of relapse rates to be expected after treatment using task-sharing methods.

Our study has limitations. Scale-up costs were estimated using anticipated rather than actual expenditures incurred; actual costs were not available from the relevant funding organisation. While actual costs could have deviated from our assumptions, we note that our core findings around the NNT value are relatively insensitive to a substantial increase in assumed costs of scale-up (figure 2). The analysis we present is also ‘short-run’ in nature, limited to a consideration of the cost-effectiveness of a programme operating at a scale of activity limited by both geographical coverage (three cities) and the resource constraints of health clinics included in the initial scale-up programme; LHWs trained in the delivery of the FB are multitasking practitioners who work across other health promotional activities competing for their time. Subsequently we do not consider the ‘long-run’ cost implications of larger expansions in treatment coverage to levels that would require investment in further LHW capacity locally, or the additional costs of extension to national coverage.

Our modelling did not allow for recurrent treatment episodes or variability in treatment response according to service user characteristics (eg, chronicity of illness). The uncertainty this introduces to our main conclusions is difficult to assess. Our sensitivity analysis was ‘deterministic’ rather than ‘probabilistic’, and as such we did not account for all aspects of uncertainty simultaneously, including sampling uncertainty relating to the key parameters of relevance. Finally, we did not evaluate wider health system resource impacts arising from the scale-up of the FB. Additional (unreported) analysis of data from the FB clinical trial showed that wider health professional contacts were infrequent, with only small differences in mean costs of service contacts between the trial arms and between participants who either scored ≥9 or <9 on the SSQ at 6 months. The main trial analysis also reports that fewer patients receiving FB treatment were referred for antidepressant medication. Taken to together, these findings suggest that modelling of wider service use effects would have been unlikely to change our main conclusions.

Our study also has strengths. This is the first attempt at assessing the cost-effectiveness of investing in the scale-up of a task-sharing model for treating CMD within routine practice in an LMIC. We anchored our modelling to evidence on treatment effectiveness derived from the same geographical and service context within which scale-up of the FB took place and our assessment of costs used data directly relating to a major programme scale-up initiative. Our analysis has also been pragmatic in considering the uncertainties and risks to achieving a cost-effective scale-up and linking cost-effectiveness to treatment coverage levels, a key implementation outcome relevant to the cost-effectiveness of scale-up and a measurable outcome for services.

## Clinical implications

The economic case for the scale-up of the FB appears convincing in principle based on evidence from Zimbabwe and its adoption at scale in similar LMIC settings should be given serious consideration. Investing in evidence on the types of scale-up strategies likely to offer a cost-effective means of sustaining required levels of treatment coverage will help focus efforts on approaches to scale-up that optimise resources invested in programmes.

## Data Availability

Data are available upon reasonable request. Data are available on reasonable request.

## References

[1] Araya R , Flynn T , Rojas G , et al . Cost-Effectiveness of a primary care treatment program for depression in low-income women in Santiago, Chile. Am J Psychiatry 2006;163:1379–87. 10.1176/ajp.2006.163.8.1379 16877650

[2] Patel V , Weobong B , Weiss HA , et al . The healthy activity program (HAP), a lay counsellor-delivered brief psychological treatment for severe depression, in primary care in India: a randomised controlled trial. Lancet 2017;389:176–85. 10.1016/S0140-6736(16)31589-6 27988143 PMC5236064

[3] Abas M , Bowers T , Manda E , et al . 'Opening up the mind': problem-solving therapy delivered by female lay health workers to improve access to evidence-based care for depression and other common mental disorders through the Friendship bench project in Zimbabwe. Int J Ment Health Syst 2016;10:39. 10.1186/s13033-016-0071-9 27175215 PMC4865009

[4] Chibanda D , Weiss HA , Verhey R , et al . Effect of a primary care-based psychological intervention on symptoms of common mental disorders in Zimbabwe: a randomized clinical trial. JAMA 2016;316:2618–26. 10.1001/jama.2016.19102 28027368

[5] Chibanda D , Verhey R , Munetsi E , et al . Scaling up interventions for depression in sub-Saharan Africa: lessons from Zimbabwe. Glob Ment Health 2016;3:e13. 10.1017/gmh.2016.8 PMC5314736 28596882

[6] Wagenaar BH , Hammett WH , Jackson C , et al . Implementation outcomes and strategies for depression interventions in low- and middle-income countries: a systematic review. Glob Ment Health 2020;7:e7. 10.1017/gmh.2020.1 PMC7176918 32346482

[7] Cubillos L , Bartels SM , Torrey WC , et al . The effectiveness and cost-effectiveness of integrating mental health services in primary care in low- and middle-income countries: systematic review. BJPsych Bull 2021;45:40–52. 10.1192/bjb.2020.35 32321610 PMC8058938

[8] Siskind D , Araya R , Kim J . Cost-effectiveness of improved primary care treatment of depression in women in Chile. Br J Psychiatry 2010;197:291–6. 10.1192/bjp.bp.109.068957 20884952

[9] Buttorff C , Hock RS , Weiss HA , et al . Economic evaluation of a task-shifting intervention for common mental disorders in India. Bull World Health Organ 2012;90:813–21. 10.2471/BLT.12.104133 23226893 PMC3506405

[10] Fenwick E , Claxton K , Sculpher M . The value of implementation and the value of information: combined and uneven development. Med Decis Making 2008;28:21–32. 10.1177/0272989X07308751 18263559

[11] Thompson C , Pulleyblank R , Parrott S , et al . The cost-effectiveness of quality improvement projects: a conceptual framework, checklist and online tool for considering the costs and consequences of implementation-based quality improvement. J Eval Clin Pract 2016;22:26–30. 10.1111/jep.12421 26201387

[12] Adam T . Making choices in health: WHO guide to cost-effectiveness analysis. World Health Organization, 2003.

[13] Machado MO , Veronese N , Sanches M , et al . The association of depression and all-cause and cause-specific mortality: an umbrella review of systematic reviews and meta-analyses. BMC Med 2018;16:1–13. 10.1186/s12916-018-1101-z PMC6053830 30025524

[14] Woods B , Revill P , Sculpher M , et al . Country-level cost-effectiveness thresholds: initial estimates and the need for further research. Value in Health 2016;19:929–35. 10.1016/j.jval.2016.02.017 27987642 PMC5193154

[15] World Bank national accounts data, and OECD national accounts data files, 2021. World Bank. Available: https://data.worldbank.org/indicator/NY.GNP.MKTP.CD?locations=ZW [Accessed July 1st, 2021].

[16] Ochalek J , Lomas J , Claxton K . Cost per DALY averted thresholds for low-and middle-income countries: evidence from cross-country data. CHE Research Paper 122 2016;2015.

[17] Ochalek J , Lomas J , Claxton K . Estimating health opportunity costs in low-income and middle-income countries: a novel approach and evidence from cross-country data. BMJ Glob Health 2018;3:e000964. 10.1136/bmjgh-2018-000964 PMC6231096 30483412

[18] Briggs A , Sculpher M , Claxton K . Decision modelling for health economic evaluation: Oup Oxford, 2006.

[19] Jelsma J , Hansen K , de Weerdt W , et al . How do Zimbabweans value health states? Popul Health Metr 2003;1:1–10. 10.1186/1478-7954-1-11 14678566 PMC317383

[20] Herdman M , Gudex C , Lloyd A , et al . Development and preliminary testing of the new five-level version of EQ-5D (EQ-5D-5L). Quality of Life Research 2011;20:1727–36. 10.1007/s11136-011-9903-x 21479777 PMC3220807

[21] Patel V , Simunyu E , Gwanzura F , et al . The Shona symptom questionnaire: the development of an Indigenous measure of common mental disorders in Harare. Acta Psychiatr Scand 1997;95:469–75. 10.1111/j.1600-0447.1997.tb10134.x 9242841

[22] Ali S , Rhodes L , Moreea O , et al . How durable is the effect of low intensity CBT for depression and anxiety? remission and relapse in a longitudinal cohort study. Behav Res Ther 2017;94:1–8. 10.1016/j.brat.2017.04.006 28437680

[23] Patel V , Todd C , Winston M , et al . Outcome of common mental disorders in Harare, Zimbabwe. Br J Psychiatry 1998;172:53–7. 10.1192/bjp.172.1.53 9534833

[24] Global Health Observatory, 2021. World Health Organisation. Available: https://www.who.int/data/gho [Accessed July 1st, 2021].

[25] Walker ER , McGee RE , Druss BG . Mortality in mental disorders and global disease burden implications: a systematic review and meta-analysis. JAMA psychiatry 2015;72:334–41. 25671328 10.1001/jamapsychiatry.2014.2502 PMC4461039

[26] Turner HC , Lauer JA , Tran BX , et al . Adjusting for inflation and currency changes within health economic studies. Value in Health 2019;22:1026–32. 10.1016/j.jval.2019.03.021 31511179

[27] Kumaranayake L . The real and the nominal? making Inflationary adjustments to cost and other economic data. Health Policy Plan 2000;15:230–4. 10.1093/heapol/15.2.230 10837047

[28] Organisation WH . Macro economics and health: investing in health for economic development, 2001.

[29] Husereau D , Drummond M , Petrou S , et al . Consolidated health economic evaluation reporting standards (cheers) statement. Int J Technol Assess Health Care 2013;29:117–22. 10.1017/S0266462313000160 23587340

