# Supplementary material for: Economic threshold analysis of delivering a task-sharing treatment for common mental disorders at scale: the Friendship Bench, Zimbabwe
Source: Evid Based Ment Health. 2021 Nov 18;25(2):47–53. doi: 10.1136/ebmental-2021-300317 (PMC9046737; doi:10.1136/ebmental-2021-300317)

Modelling YLDs/DALYs

This is a cohort-based Markov state transition model. The blue bubbles represent health states (CMD, remission or death) characterised by a unique disability weight (disability weight for death=1). The straight arrows represent transitions between states occurring with a given probability (or in this case the % of the model cohort who transition as shown); the curved arrows indicate that probability of remaining in a specific state.

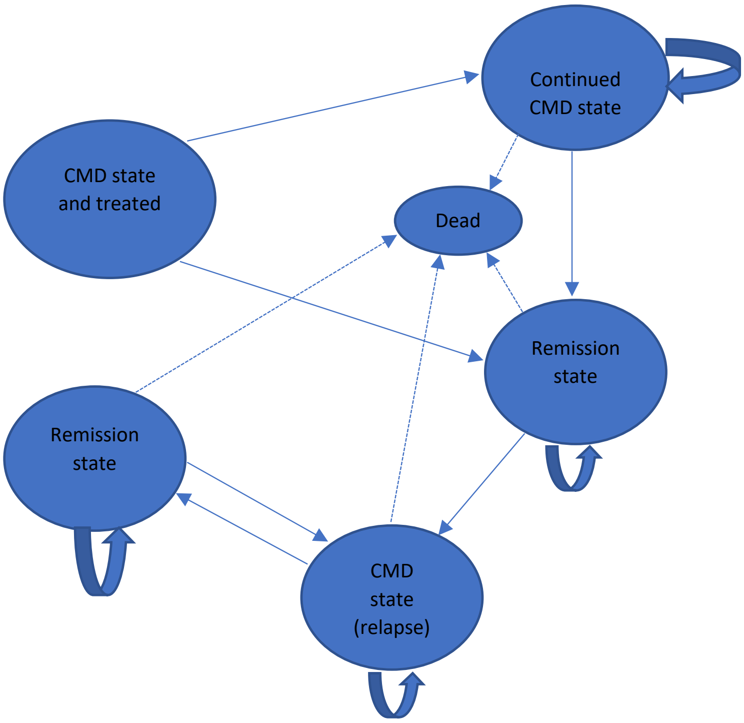

Supplement: Supplementary data [file ebmental-2021-300317supp001.pdf]
